# High posterior tibial slope is associated with higher failure rates in nonoperative management of primary anterior cruciate ligament injury

**DOI:** 10.1002/jeo2.70502

**Published:** 2025-10-30

**Authors:** Svenja Heidecke, Nikolaus Kraml, David Haslhofer, Paul M. Schwarz, Tobias Gotterbarm, Philipp W. Winkler

**Affiliations:** ^1^ Department of Orthopaedics and Traumatology, Kepler University Hospital GmbH Johannes Kepler University Linz Linz Austria

**Keywords:** ACL, bony morphology, conservative treatment, cruciate ligament, failure, revision

## Abstract

**Purpose:**

To evaluate the impact of posterior tibial slope (PTS) on patient‐reported outcome measures (PROMs) and treatment failure in patients with primary anterior cruciate ligament (ACL) injury who elected to undergo nonoperative treatment.

**Methods:**

Patients with primary ACL injury who underwent nonoperative treatment were included in this retrospective study. A chart review was conducted to collect demographic data. Medial PTS was measured on strict lateral radiographs using a standardised method. Validated PROMs were collected at final follow‐up. A correlation analysis was conducted to assess the relationship between PTS and PROMs. A logistic regression analysis was performed to assess whether PTS could predict failure of nonoperative treatment. Failure was defined as conversion to ACL reconstruction after a minimum of 3 months of nonoperative treatment.

**Results:**

A total of 113 patients with a mean age of 31.7 ± 10.5 years at the time of ACL injury and a mean follow‐up period of 6.6 ± 1.5 years were included in this study. Nonoperative treatment failure occurred in 82 patients (73%) after a median time of 10.0 months (interquartile range, 7 months) from injury. Patients experiencing nonoperative treatment failure showed a significantly higher PTS compared to patients without treatment failure (10.5° ± 2.8° vs. 8.1° ± 2.1°; *p* < 0.05). Thirty‐two per cent of patients with nonoperative treatment failure had a PTS ≥ 12°, while 100% of patients without nonoperative treatment failure had a PTS < 12° (*p* < 0.001). Each one‐degree increase in PTS was associated with a 1.5‐fold increase in the odds of nonoperative treatment failure (*p* < 0.05) after primary ACL injury. No significant correlations were observed between PTS and PROMs.

**Conclusions:**

A high PTS was associated with an increased risk of nonoperative treatment failure in primary ACL injury and often required delayed ACL reconstruction. However, PTS was not an independent predictor. Treatment decisions should also consider established factors such as patient age and activity level.

**Level of Evidence:**

Level III, retrospective case series.

**Abbreviations**
ACL: anterior cruciate ligament
ACL‐RSI: ACL‐return to sport after injury scale
ADL: activities of daily living
IKDC‐SKF: International Knee Documentation Committee Subjective Knee Form
KOOS: knee injury and osteoarthritis outcome score
MPTS: medial posterior tibial slope
NRS: numeric rating scale
PROMs: patient‐reported outcome measures
PTS: posterior tibial slope
QOL: knee‐related quality of life
SEM: standard error of measurement
Sport/Rec: sport and recreation function
TAS: Tegner activity scale

## INTRODUCTION

The treatment of primary anterior cruciate ligament (ACL) injury remains a topic of ongoing debate [7]. Although some patients may benefit from nonoperative management, there is a considerable risk of treatment failure. In a randomised controlled trial, 39% of patients initially randomised to nonoperative treatment for a primary ACL injury eventually underwent delayed ACL reconstruction, highlighting the substantial failure rate of nonoperative management [11]. ACL reconstruction not only restores knee stability but also seeks to optimise functional outcomes and prevent secondary knee injuries [3, 24, 25]. Both modifiable and nonmodifiable risk factors influencing the treatment success of primary ACL injury have been reported, including age, activity level, bony morphology and concomitant injuries [7]. Ideally, the treatment of a primary ACL injury should be tailored to the patient′s risk profile and functional demands [14].

In recent years, numerous studies in ACL research have focused on the posterior tibial slope (PTS). Clinical and biomechanical research suggests that an increased PTS alters knee kinematics and increases forces acting on both the native and reconstructed ACL [1, 2, 13, 39]. Consequently, a high PTS has been shown to be associated with primary [18] and recurrent ACL injury [2, 38]. Although a high PTS has been associated with an increased risk of ACL injury and ACL graft failure, its clinical relevance remains controversial, as high‐quality studies have yielded conflicting results [17, 39].

Currently, only limited evidence exists on how PTS influences the success of nonoperative management in patients with primary ACL injuries. One study with a small cohort (*N* = 37) investigated the association between PTS and failure of nonoperative management in patients with ACL injuries [29]. Clinical outcomes were not collected, leading to limited evidence regarding the impact of PTS on the success of nonoperative treatment in ACL injuries.

The purpose of the present study was to assess the impact of PTS on patient‐reported outcome measures (PROMs) and treatment failure in patients with primary ACL injury who elected to undergo nonoperative treatment. It was hypothesised that PTS is negatively correlated with PROMs and that a high PTS increases the risk of failure of nonoperative management in patients with primary ACL injuries.

## MATERIALS AND METHODS

Study approval was given by the local ethics committee (No.: 1143/2023). Written informed consent was obtained from all included patients. This retrospective case series included 273 patients diagnosed with primary ACL injury between January 2014 and December 2019, all of whom elected to undergo nonoperative treatment. The inclusion criteria were as follows: (1) Age between 18 and 60 years at the time of primary ACL injury; (2) Magnetic resonance imaging (MRI) showing complete ACL rupture; (3) Available strict lateral knee radiographs; (4) Patients who elected to undergo nonoperative treatment; (5) Nonoperative management including physiotherapy and weight‐bearing as tolerated for a minimum of 3 months; (6) Minimum follow‐up of 24 months for patients treated nonoperatively; (7) Minimum follow‐up of 3 months for patients with treatment failure (i.e., conversion to ACL reconstruction). The exclusion criteria were as follows: (1) History of previous ipsilateral or contralateral ACL injury; (2) Osteoarthritis > Grade 2 according to Kellgren–Lawrence Scale at the time of ACL injury; (3) Posttraumatic deformity of the lower extremity; (4) Previous periarticular fractures or osteotomies around the knee joint; (5) Concomitant ligamentous injuries; (6) Primary operative management; (7) Missing medical or radiological documentation; (8) Concomitant injuries (i.e., meniscus, cartilage etc.) requiring surgical treatment.

### Demographic and injury‐related data

A chart review was conducted between March 2023 and September 2023 to collect demographic and injury‐related data. Patient‐specific data included date of birth, age at the time of injury, sex, laterality, previous injuries and subsequent knee surgeries. The following injury‐related data were recorded: date of injury, time from injury to treatment failure (i.e., ACL reconstruction) and concomitant injuries.

### Nonoperative management

Patients opting for nonoperative management of primary ACL injury were recommended to undergo physiotherapy sessions one or two times per week for a minimum of 3 months after injury. The aims of the physiotherapy were to restore full range of motion, strength and neuromuscular training. Physiotherapy was performed in various institutions. Weight‐bearing as tolerated was recommended from day one. A knee brace was not recommended. Nonsteroidal anti‐inflammatory drugs were recommended to relieve pain and inflammation.

### Treatment failure

Failure of nonoperative treatment was defined as conversion from nonoperative treatment to operative treatment (i.e., ACL reconstruction) due to persistent instability or pain after a minimum of 3 months of nonoperative treatment.

### PTS measurement

**Figure 1:**
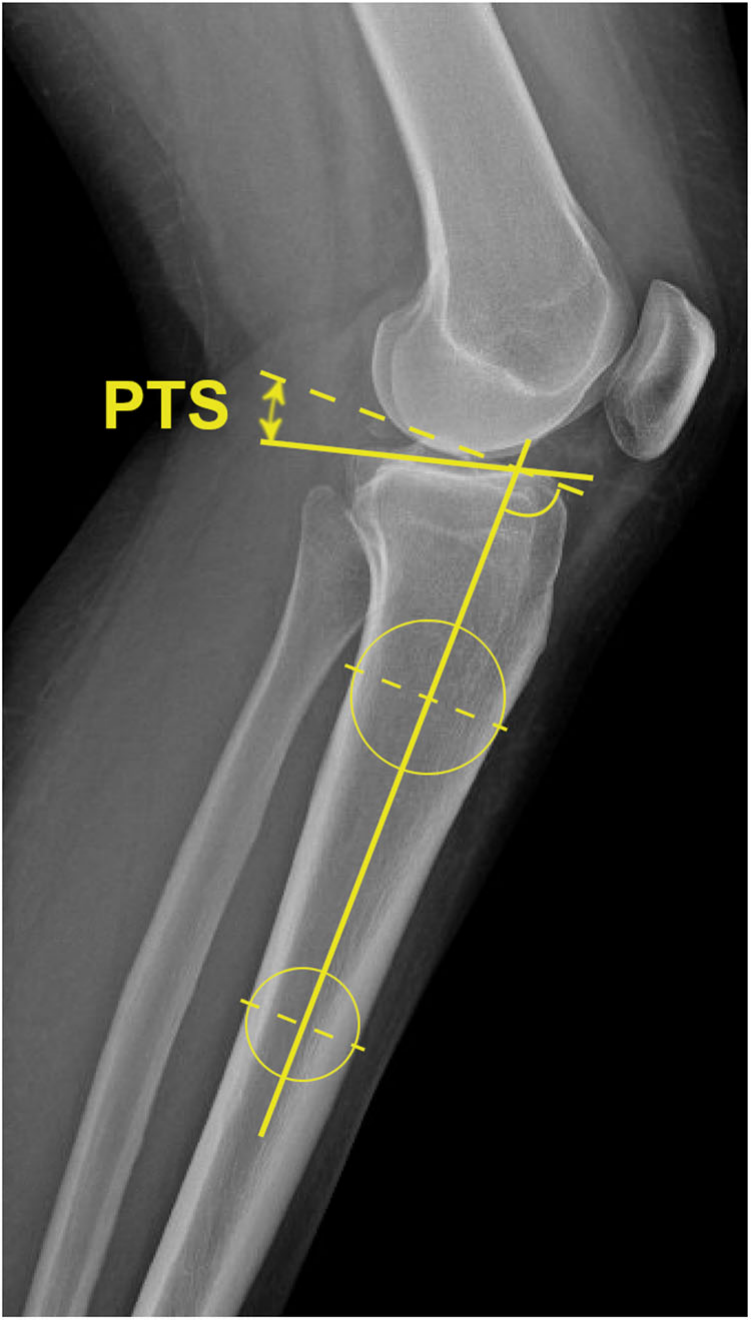
Medial posterior tibial slope (PTS) measurement. PTS measurement is shown in a left knee demonstrating a medial PTS of 16.7°.

To ensure accurate measurement of the medial PTS, true lateral radiographs were used. Radiographs with a femoral condyle overlap exceeding 6 mm were excluded to avoid inaccuracies associated with malrotated radiographs [31, 36]. The medial PTS was determined using a standardised technique [6]. The proximal tibial shaft axis was defined by drawing a line connecting the centres of two circles positioned 5 and 15 cm distal to the joint line, with the circles tangential to the anterior and posterior cortices of the tibia. A tangential line was then drawn along the medial tibial plateau. The medial PTS was calculated by subtracting the angle between the proximal tibial shaft axis and the tangential line from the medial tibial plateau from 90° (Figure 1). Measurements of the PTS were performed by observer 1 (S.H.) using DeepUnity Diagnost 2.0.2.1 (DH Healthcare GmbH, DE). To assess the intra‐ and interrater reliability, intraclass correlation coefficients (ICC) and the standard error of measurement (SEM) were calculated. For this purpose, 20 lateral knee radiographs were randomly selected, and the medial PTS was measured three times by observer 1 (S.H.) at 2‐week intervals and once by observer 2 (N.K.). The results showed excellent intrarater reliability (ICC: 0.97, 95% confidence interval [CI]: [0.927, 0.99]; SEM, 0.49°) and good to excellent interrater reliability (ICC: 0.96, 95% CI [0.84, 0.99]; SEM, 0.56°).

### PROMs

Standardised, German‐validated PROMs were sent to eligible patients. These included the International Knee Documentation Committee Subjective Knee Form (IKDC‐SKF), the knee injury osteoarthritis outcome score (KOOS), the Lysholm Score, the Tegner Activity Scale (TAS), the ACL return to sport after injury scale (ACL‐RSI) and numeric rating scales (NRS) for pain and instability [22, 28, 32, 40, 41].

### Statistical analysis

The required sample size was calculated a priori based on PTS. A mean difference of 1.4° was considered clinically relevant, based on previous findings by Webb et al. [37]. A standard deviation of 2.3° for the PTS was used for sample size calculation [37]. Accordingly, a total sample size of 96 patients was required to achieve a statistical power of 0.80 (failure group: *n* = 48; nonfailure group: *n* = 48; effect size: 0.61; significance level: 0.05). The power analysis was conducted using G*Power (Erdfelder, Faul, Buchner, Lang, HHU Düsseldorf).

Statistical analysis was performed with SPSS software version 30.0 (IBM‐SPSS), with the level of significance set at *α* = 0.05. Categorical variables were expressed as counts with corresponding percentages. Based on the data distribution, assessed using the Shapiro–Wilk test, continuous variables were reported as mean ± standard deviation for normally distributed data or as median and interquartile range (IQR) for nonnormally distributed data. Spearman's rank‐order correlation was employed to examine the relationship between medial PTS and PROMs, including the IKDC‐SKF, KOOS subscales, Lysholm Score, TAS, ACL‐RSI and NRS for pain and instability in patients undergoing nonoperative treatment of ACL injury. Comparisons of categorical variables between groups were conducted using Fisher′s exact test, while continuous variables were compared using either the Mann–Whitney *U* test or the unpaired *t*‐test, depending on distribution. Additionally, a binomial logistic regression model was used to investigate whether medial PTS (independent variable) could predict failure of nonoperative treatment (dependent variable).

## RESULTS

**Figure 2:**
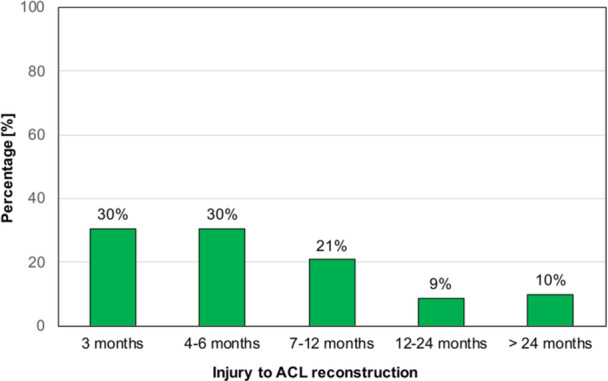
Histogram illustrating the proportion of patients who underwent ACL reconstruction within specific timeframes after ACL injury. ACL, anterior cruciate ligament.

A total of 273 patients were screened for eligibility. One hundred sixty patients were excluded for the following reasons: lost to follow‐up (*n* = 94), incomplete medical records (*n* = 42), partial ACL rupture (*n* = 13), previous ACL injury (*n* = 4), contralateral ACL injury (*n* = 2), lack of interest in participating (*n* = 2), concomitant posterior cruciate ligament injury (*n* = 1), ACL rupture in childhood (*n* = 1) and chronic ACL insufficiency (*n* = 1). Therefore, a total of 113 patients (41%) met the inclusion criteria. Patient‐ and injury‐related characteristics of the total study group are shown in Table 1. The mean age at the time of ACL injury was 31.7 ± 10.5 years (range, 18–53 years). In 82 (73%) patients, nonoperative treatment failed and ACL reconstruction was performed at a median time of 10.0 months (IQR: 7.0 months) after ACL injury. A histogram illustrating the proportion of patients who underwent ACL reconstruction within specific timeframes after ACL injury is shown in Figure 2. Consequently, the nonoperative treatment failure group comprised 82 patients with a mean follow‐up of 24 ± 30.5 months, while the nonfailure group included 31 patients with a mean follow‐up of 71 ± 24.5 months (*p* < 0.001, Table 2).

**Table 1:** Patient‐ and Injury‐related characteristics.

Variable	Total study group
Number of included patients, *n*	113
Follow‐up (months)	37.0 ± 35.8 (3–112)
Age,^a^ (years)	31.7 ± 10.5 (18–53)
Males, *n* (%)	71 (63%)
Right knee, *n* (%)	59 (52%)
Concomitant meniscus injury	
None, *n* (%)	60 (53%)
Medial, *n* (%)	33 (29%)
Lateral, *n* (%)	11 (10%)
Medial + Lateral, *n* (%)	9 (8%)
MPTS (°)	9.9 ± 2.8 (3.4–18.7)
IKDC‐SKF	82.2 ± 17.1 (21.8–100)
KOOS	
Symptoms	84.9 ± 16.8 (39.3–100)
Pain	84.9 ± 13.9 (50.0–100)
ADL	93.2 ± 12.9 (55.9–100)
Sport/Rec	76.3 ± 26.5 (10–100)
QOL	72.6 ± 27.1 (12.5–100)
Lysholm	86.0 ± 16.1 (32.0–100)
Tegner activity scale^b^	5 (3)
ACL‐RSI	63.3 ± 29.0 (0‐100)
NRS (pain)	1.3 ± 1.6 (0–6)
NRS (instability)	1.5 ± 2.1 (0–9)

*Note*: Categorical variables are expressed as count (percentage). Continuous variables are expressed as mean ± standard deviation (range), unless otherwise noted.

Abbreviations: ACL‐RSI, ACL‐return to sport after injury scale; ADL, activities of daily living; IKDC‐SKF, International Knee Documentation Committee Subjective Knee Form; KOOS, knee injury and osteoarthritis outcome score; MPTS, medial posterior tibial slope; NRS, numeric rating scale; PROMs, patient‐reported outcome measures; QOL, knee‐related quality of life; Sport/Rec, sport and recreation function.

^a^ Age at the time of ACL injury.

^b^ Median (interquartile range); PROMs were available for 41 patients (36%).

**Table 2:** Patients with and without nonoperative treatment failure.

Variable	Failure (*n* = 82)	Nonfailure (*n* = 31)	*p* value
Follow‐up (months)	24 ± 30.5 (3–108)	71 ± 24.5 (24–112)	<0.001
Injury to ACL‐R (months)	10 ± 12.8 (3–79)	–	–
Age,^a^ (years)	30.3 ± 10 (16–52)	35.2 ± 11 (16–53)	0.028
Males, *n* (%)	55 (67%)	16 (52%)	n.s.
Right knee, *n* (%)	42 (51%)	17 (55%)	n.s.
Concomitant meniscus injury			n.s.
None, *n* (%)	44 (54%)	16 (52%)	
Medial, *n* (%)	21 (26%)	12 (39%)	
Lateral, *n* (%)	10 (12%)	1 (3%)	
Medial + Lateral, *n* (%)	7 (9%)	2 (7%)	
MPTS, (°)	10.5 ± 2.8 (5.9–18.7)	8.1 ± 2.1 (5.0–11.8)	<0.001
MPTS ≥ 12°	26 (32%)	0 (0%)	<0.001
PROMs available	16 (20%)	25 (81%)	
IKDC‐SKF	81.1 ± 14.7 (55.2–100)	82.90 ± 18.8 (21.8–100)	n.s.
KOOS			
Symptoms	83.48 ± 14.3 (53.6–100)	85.85 ± 18.4 (39.3–100)	n.s.
Pain	89.7 ± 11.8 (69.4–100)	89.09 ± 15.3 (50–100)	n.s.
ADL	92.36 ± 14.0 (55.9–100)	93.70 ± 12.3 (57.4–100)	n.s.
Sport/Rec	76.56 ± 25.3 (30–100)	76.2 ± 27.7 (10–100)	n.s.
QOL	71.48 ± 26.7 (25–100)	73.3 ± 27.8 (12.5–100)	n.s.
Lysholm	86.87 ± 11.4 (62.0–100)	85.4 ± 18.8 (32.0–100)	n.s.
Tegner activity scale^b^	6 (3)	4 (3)	0.011
ACL‐RSI	62.09 ± 21.6 (22.5–96.7)	64.03 ± 33.3 (0–100)	n.s.
NRS (Pain)	1.4 ± 1.8 (0–6)	1.2 ± 1.5 (0–5)	n.s.
NRS (Instability)	1.3 ± 1.6 (0–4)	1.6 ± 2.3 (0–9)	n.s.

*Note*: Categorical variables are expressed as count (percentage). Continuous variables are expressed as mean ± standard deviation (range), unless otherwise noted.

Abbreviations: ACL‐RSI, ACL‐return to sport after injury scale; ADL, activities of daily living; IKDC‐SKF, International Knee Documentation Committee Subjective Knee Form; KOOS, knee injury and osteoarthritis outcome score; MPTS, medial posterior tibial slope; n.s., nonsignificant (*p* > 0.05); NRS, numeric rating scale; PROMs, patient‐reported outcome measures; QOL, knee‐related quality of life; Sport/Rec, sport and recreation function.

^a^ Age at the time of ACL injury.

^b^ Median (interquartile range).

**Figure 3:**
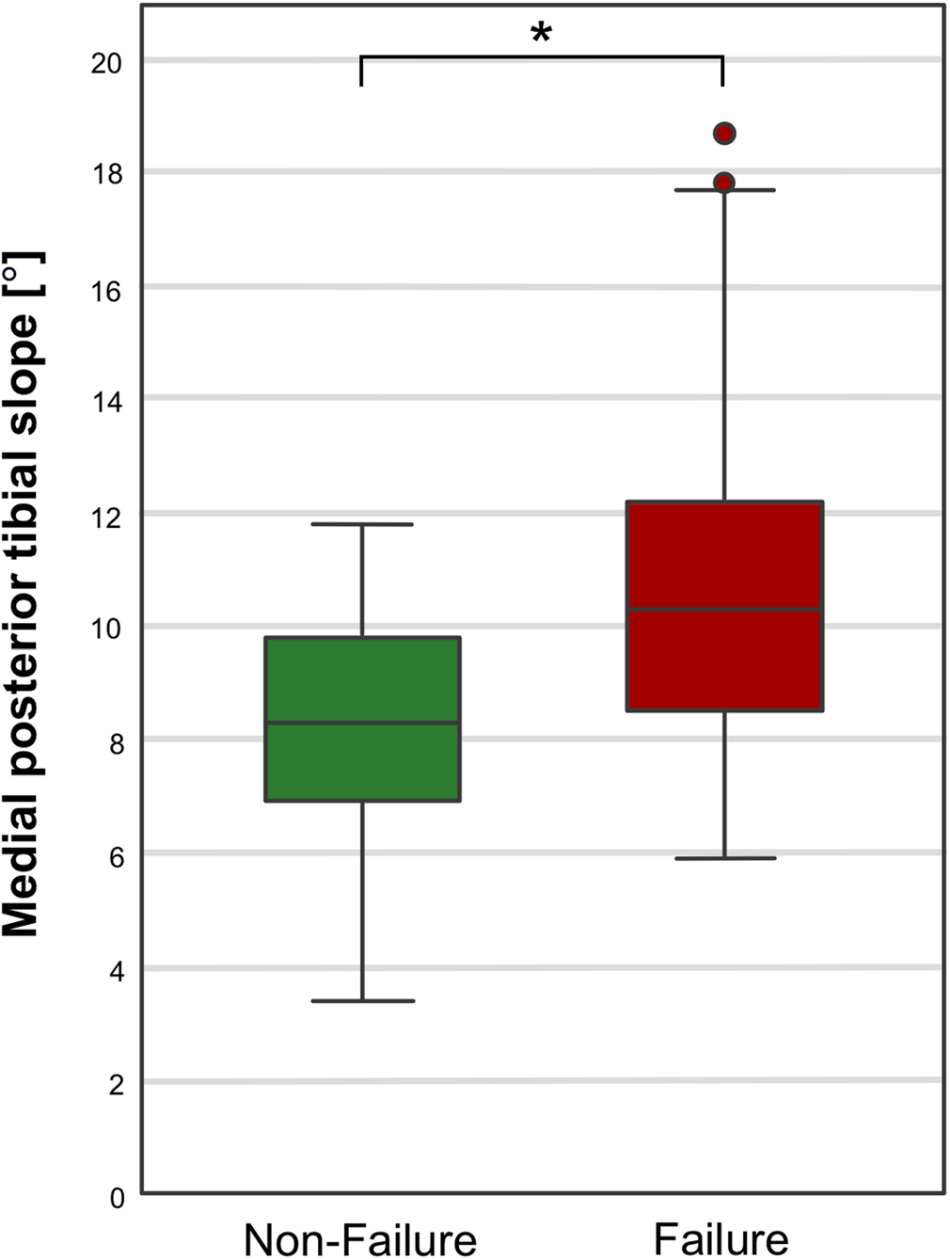
Medial PTS in patients with and without nonoperative treatment failure. A statistically significant difference in medial posterior tibial slope (PTS) between patients with and without nonoperative treatment failure was detected (*).

The mean medial PTS for the entire study cohort was 9.9 ± 2.8° (range, 3°–19°). A statistically significant difference in medial PTS was found between the nonoperative treatment failure group (10.5° ± 2.8°) and the nonfailure group (8.1° ± 2.1°, *p* < 0.01, Figure 3). In the nonoperative treatment failure group, 26 (32%) patients had a PTS ≥ 12°, whereas in the nonfailure group, all patients (*n* = 31, 100%) had a PTS < 12° (*p* < 0.001). Concomitant meniscus injuries were observed in 53 patients (47%) with no between group difference (*p* > 0.05). Detailed information between the nonoperative treatment failure group and the nonfailure group are shown in Table 2.

Group comparison demonstrated significantly higher scores on the TAS at final follow‐up in the nonoperative treatment failure group following ACL reconstruction compared to the nonfailure group (median 6 vs. median 4).

At an average follow‐up of 6.6 ± 1.5 years (range, 2.7–9.4 years), PROMs were available for 41 patients (36%). A detailed overview of the PROMs is presented in Table 2. No statistically significant differences in PROMs were observed between the failure and nonfailure groups (all *p* > 0.05; Table 2). Spearman′s rank‐order correlation analysis revealed a statistically significant correlation between the medial PTS and the KOOS Symptoms subscale (*r_s_* = 0.446, *p* < 0.05, Table 3).

**Table 3:** Correlation analysis between patient‐reported outcome measures and medial PTS.

Patient‐reported outcome score^a^	Medial PTS
IKDC‐SKF	*r_s_* = −0.246 *p* > 0.05
KOOS	
Symptoms	*r_s_* = 0.446 *p* = 0.025
Pain	*r_s_* = −0.383 *p* > 0.05
ADL	*r_s_* = −0.306 *p* > 0.05
Sport/Rec	*r_s_* = −0.338 *p* > 0.05
QoL	*r_s_* = −0.222 *p* > 0.05
Lysholm score	*r_s_* = −0.026 *p* > 0.05
Tegner activity scale	*r_s_* = −0.109 *p* > 0.05
ACL‐RSI	*r_s_* = −0.143 *p* > 0.05
NRS (pain)	*r_s_* = 0.251 *p* > 0.05
NRS (instability)	*r_s_* = 0.218 *p* > 0.05

Abbreviations: ACL‐RSI, ACL‐return to sport after injury scale; ADL, activities of daily living; IKDC‐SKF, International Knee Documentation Committee Subjective Knee Form; KOOS, knee injury and osteoarthritis outcome score; MPTS, medial posterior tibial slope; NRS, numeric rating scale; QoL, knee‐related quality of life; Sport/Rec, sport and recreation function.

^a^ Data are available for 41 (36%) patients.

One hundred thirteen cases were included in the binomial logistic regression model, which correctly classified 74% of cases and was statistically significant (*p* < 0.05). The medial PTS was shown to be a statistically significant predictor of nonoperative treatment failure (*p* < 0.001). It was found that each increase in medial PTS by one‐degree results in a 1.5‐fold increase in the odds of treatment failure in nonoperative management of ACL injuries (95% CI: [1.21, 1.87], *p* < 0.001).

## DISCUSSION

The most important finding of this study was the association between increased medial PTS and failure of nonoperative management in patients with primary ACL injury. Each one‐degree increase in medial PTS resulted in a 1.5‐fold increase in the odds of failure of nonoperative treatment. In addition, one‐third of the patients with failed nonoperative treatment had a medial PTS ≥ 12°, while none of the patients without treatment failure had a medial PTS ≥ 12°. Therefore, ACL reconstruction should be considered as the treatment of choice in patients with a high PTS and primary ACL injury. The findings of this study contribute valuable information, but should be interpreted with caution given its retrospective nature and the possible influence of confounders such as age and activity level.

The optimal treatment for patients with primary ACL injury remains a topic of ongoing debate [7]. The conflicting study results do not permit a single treatment recommendation that fits every patient [7, 26, 34]. Nevertheless, previous studies have shown, that nonoperative management of ACL injuries increase the risk of posttraumatic knee osteoarthritis [12, 16, 30]. Although posttraumatic knee osteoarthritis was also found after ACL reconstruction, this should still be evaluated in the future using modern ACL reconstruction techniques [21, 23, 42]. Other risks of nonoperative management of ACL injuries are secondary meniscal [23, 34] and cartilage injuries [30] and lower activity levels in sports and daily life [12, 16]. In this study, PROMs were satisfactory and were comparable to those of surgically treated patients [12, 16, 42].

As demonstrated in a randomised controlled trial, nonoperative treatment for primary ACL injury commonly fails, with nearly 40% of patients ultimately requiring delayed ACL reconstruction [11]. In particular, young patients [8, 20] and patients with a high preinjury activity level [10, 14, 20] have an increased risk of failure of nonoperative management, mainly due to instability and pain during physical activity [33]. A similar finding was observed in this study, where patients who experienced nonoperative treatment failure were younger and physically more active than patients without failure of nonoperative management. According to a recent expert consensus on the treatment of ACL injuries, 96% of experts agreed that individual anatomical factors, such as PTS and femoral condyle morphology, should also be considered when recommending operative or nonoperative treatment [7]. This study underscores the importance of PTS in treatment decision‐making after primary ACL injury.

The present study showed a high failure rate of nonoperative treatment reaching 73%. In line with previous studies, young patient age was associated with an increased risk of failure of nonoperative management in this study [8, 14]. While age has traditionally been considered a key factor in ACL injury management, recent reports indicate that structural characteristics, such as PTS, may be more predictive of treatment outcomes than chronological age alone [43, 44]. This study identified the medial PTS as a risk factor for nonoperative treatment failure. One‐third of the failure group had a PTS ≥ 12°, suggesting that a steeper PTS may contribute to an increased risk of failure in nonoperative management. Therefore, the medial PTS should be considered in patient counseling regarding operative and nonoperative treatment after ACL injury.

The PTS as a risk factor for primary ACL injury [18] and re‐injury after ACL reconstruction have already been described [15, 33, 38]. However, other studies did not reach the same conclusion and found no correlation between increased PTS and failure of operative management [4, 35]. Particularly in the context of anatomic ACL reconstruction, the PTS does not appear to affect the outcome [17]. Nonetheless, while an increased PTS may exert limited influence on outcomes following anatomic ACL reconstruction, its biomechanical relevance appears to be considerably greater in ACL‐deficient knees. In the absence of a functional ACL, the PTS directly contributes to anterior tibial translation, particularly under axial loading or during dynamic activities [5, 19]. This anterior shift of the tibia relative to the femur increases the mechanical demands placed on secondary stabilising structures, such as the medial meniscus. In ACL‐deficient knees, the posterior horn of the medial meniscus resists anterior tibial translation and is therefore subjected to increased biomechanical loading and stress [27]. Furthermore, a high PTS can amplify pivoting forces and rotational instability [9], which are difficult to control through nonoperative measures alone. The evaluation of the PTS in nonoperative treatment of ACL injuries has gained limited attention so far. Only one study with a small sample size and a short follow‐up has been conducted, and no PROMs have been collected. In that retrospective study, 37 patients were included, all treated with a standardised treatment protocol including knee bracing and rehabilitation. Patients were categorised based on treatment outcome into a nonoperative group and a failure group requiring surgery. A significantly lower mean PTS was observed in the group with successful nonoperative treatment (8.3° vs. 10.2°), suggesting an association between increased PTS and failure of nonoperative management. However, several limitations of that study need to be acknowledged. Besides the small sample size and lack of PROMs, there was no long‐term follow‐up to evaluate treatment outcomes and assess joint stability. At the last follow‐up (after 6 months of nonoperative treatment), only functional tests including the Lachman test, pivot shift test, and telos stress radiographs were performed, which did not always correlate with the patients' subjective perception of knee stability or clinical symptoms [29]. The results are consistent with the present study that patients who undergo delayed ACL reconstruction because of failure of nonoperative management have a significantly higher PTS compared to those who achieve successful nonoperative treatment. In fact, all patients in the present study with a slope ≥12° experienced failure of nonoperative treatment. Similarly, no patient with an increased PTS was successfully treated nonoperatively in the study by Park et al. [29]. Therefore, nonoperative management after primary ACL injury should not be recommended for patients with a high PTS.

There are several limitations to the study. First, the small sample size may have limited the statistical power of the study. While a statistically significant difference was observed for the analysis of failure of nonoperative management, the analysis of the association between PTS and PROMs did not show statistically significant differences. The analysis on the impact of PTS on PROMs may have been underpowered. Therefore, these findings should be interpreted with caution. Additionally, the high drop‐out rate could introduce selection bias and may limit the generalisability of the results. Although no active patient selection was performed, selection bias need to be considered in this retrospective study. Another limitation is that no multivariate regression model was used to adjust for confounders such as age, sex, activity level, or concomitant injuries, which may have influenced treatment outcomes independently of PTS. Additionally, the failure group was on average younger than the nonfailure group, which may have introduced a confounding effect on treatment outcomes that was not adjusted for. Longer follow‐up periods could provide further insight into the long‐term implications of PTS on outcomes after nonoperative management of primary ACL injury. Another limitation was that physiotherapy was performed at various institutions and did not follow a standardised protocol.

## CONCLUSIONS

A high PTS was associated with an increased risk of failure of nonoperative management in patients with primary ACL injury and often required delayed ACL reconstruction. However, PTS was not an independent predictor. Treatment decisions should also consider established factors such as patient age and activity level.

## AUTHOR CONTRIBUTIONS

All listed authors have contributed substantially to this work: Svenja Heidecke, Nikolaus Kraml, David Haslhofer, Paul M. Schwarz and Philipp W. Winkler collected data, performed statistical analysis, literature review and primary manuscript preparation. Tobias Gotterbarm, Paul M. Schwarz and Philipp W. Winkler assisted with interpretation of the results as well as editing and final manuscript preparation. All authors read and approved the final manuscript.

## CONFLICT OF INTEREST STATEMENT

Philipp W. Winkler works as Web Editor for Knee Surgery Sports Traumatology Arthroscopy (KSSTA). Tobias Gotterbarm: Grant and personal fees (Zimmer Biomet Europe), Depuye Synthes GmbH, Mathys AG), personal fees (Medacta, ImplanTec). The remaining authors declare no conflict of interest.

## ETHICS STATEMENT

This study was approved by the Ethics Committee of the Medical Faculty, Johannes Kepler University (No.: 1143/2023). Written informed consent was obtained from each patient who completed the questionnaire of this study.

## Data Availability

Data are available from the corresponding author upon reasonable request.
